# Characterization of Mesenchymal Stem Cells Derived from Patients with Cerebellar Ataxia: Downregulation of the Anti-Inflammatory Secretome Profile

**DOI:** 10.3390/cells9010212

**Published:** 2020-01-15

**Authors:** Jong-Heon Kim, Jin Han, Donggun Seo, Jong Hyuk Yoon, Dongyeong Yoon, Jungwan Hong, Sang Ryong Kim, Min Sung Kim, Tae Yong Lee, Kyung Suk Kim, Pan-Woo Ko, Ho-Won Lee, Kyoungho Suk

**Affiliations:** 1 Brain Science & Engineering Institute, School of Medicine, Kyungpook National University, Daegu 41944, Korea; jongheonkim@knu.ac.kr (J.-H.K.); jungwan33@naver.com (J.H.); srk75@knu.ac.kr (S.R.K.); panwoo.ko@gmail.com (P.-W.K.);; 2 Department of Pharmacology and Biomedical Science, School of Medicine, Kyungpook National University, Daegu 41944, Korea; jinhan6628@gmail.com (J.H.); seodonggun3163@naver.com (D.S.); 3 Neurodegenerative Diseases Research Group, Korea Brain Research Institute, Daegu 41062, Korea; jhyoon@kbri.re.kr; 4 School of Life Sciences, BK21 plus KNU Creative BioResearch Group, Kyungpook National University, Daegu 41566, Korea; ydy10@naver.com; 5 Bioengineering Institute, Corestem Inc., Seoul 13486, Korea; mskim@corestem.com (M.S.K.); tylee@corestem.com (T.Y.L.); kskim@corestem.com (K.S.K.); 6 Department of Neurology, Kyungpook National University Chilgok Hospital, Daegu 41404, Korea

**Keywords:** antiinflammation, cerebellar ataxia, mesenchymal stem cells

## Abstract

Mesenchymal stem cell (MSC) therapy is a promising alternative approach for the treatment of neurodegenerative diseases, according to its neuroprotective and immunomodulatory potential. Despite numerous clinical trials involving autologous MSCs, their outcomes have often been unsuccessful. Several reports have indicated that MSCs from patients have low capacities in terms of the secretion of neurotrophic or anti-inflammatory factors, which might be associated with cell senescence or disease severity. Therefore, a new strategy to improve their capacities is required for optimal efficacy of autologous MSC therapy. In this study, we compared the secretory potential of MSCs among cerebellar ataxia patients (CA-MSCs) and healthy individuals (H-MSCs). Our results, including secretome analysis findings, revealed that CA-MSCs have lower capacities in terms of proliferation, oxidative stress response, motility, and immunomodulatory functions when compared with H-MSCs. The functional differences were validated in a scratch wound healing assay and neuron-glia co-cultures. In addition, the neuroprotective and immunoregulatory protein follistatin-like 1 (FSTL1) was identified as one of the downregulated proteins in the CA-MSC secretome, with suppressive effects on proinflammatory microglial activation. Our study findings suggest that targeting aspects of the downregulated anti-inflammatory secretome, such as FSTL1, might improve the efficacy of autologous MSC therapy for CA.

## 1. Introduction

Stem cell-based therapy is clinically attractive for treating various neurological disorders [1,2,3]. Among the various types of stem cells, the therapeutic potential of mesenchymal stem cells (MSCs) has gained broad attention because of their self-renewal capacity, plasticity in differentiation, neurotrophic property, and immune modulation [4,5]. MSCs are easily accessible, stable, and expandable, with few ethical issues. Although bone marrow-derived MSCs have been most widely studied [6], MSCs isolated from other tissues, such as adipose tissues or umbilical cord blood, have been developed and used [7,8,9]. Comparative studies for biological characteristics of MSCs originated from different tissues have demonstrated that they have similar multilineage differentiation potentials and gene expression profiles, but have a significant difference in bioactivities [10,11,12,13,14]. Nevertheless, bone marrow-derived MSCs are most frequently utilized [15]. More than 2000 patients have received autologous or culture-expanded allogeneic MSCs for treatment [16]. Generally, autologous MSCs are safer than allogenic MSCs. However, there are certain limitations in the use of autologous MSCs, such as aging, malignant transformation, cross-contamination, poor engraftment, limited differentiation in vivo, and inconclusive results of clinical studies [17]. MSCs from patients usually have a low capacity with regard to neurotrophic factor secretion, migration, and immunomodulation, depending on age, genetic traits, and disease severity [18,19,20]. Therefore, the challenging issue is how to improve the capacity of MSCs from patients. A new paradigm to overcome these limitations, for example, developing better culture techniques, gene-modified MSCs, preactivated MSCs, genetic modification, and MSC-based combination cell therapy, is required [21].

Cerebellar ataxia (CA) is a complex motor disorder showing the symptoms of gait instability, limb incoordination, slurred speech, and nystagmus. CA can be caused by a variety of genetic ataxias or acquired diseases, such as stroke, tumors, multiple sclerosis, paraneoplastic syndromes, and metabolic disorders [22,23]. Currently, global epidemiological studies on ataxia have reported an overall ataxia prevalence rate of 26 per 100,000 children, with a prevalence rate of 2.7 per 100,000 for dominant hereditary CA and a prevalence rate of 3.3 per 100,000 for recessive hereditary CA [24]. CA is rare, and the field of CA is highly challenging among clinicians and researchers, because the disease involves a multitude of genetic factors coupled with a variable disease progression rate and is closely related with complications caused by other neurological disorders, such as brain injury and infection [23]. There is currently no accepted cure or disease-modifying treatment for ataxia and no Food and Drug Administration-approved treatment for cerebellar motor syndrome. Considering this, MSCs might represent a new therapeutic option for CA [25,26]. Thus, it is important to understand the cellular and molecular characteristics of MSCs derived from CA patients (CA-MSCs). The present study aims to characterize CA-MSCs.

## 2. Materials and Methods

### 2.1. Cell Culture

#### 2.1.1. Human MSCs

MSCs were obtained from the bone marrow of healthy individuals and CA patients according to the reviewed and approved protocols of the Institutional Review Board of Chilgok Kyungpook National University Hospital (IRB: 2017-11-015-007). Isolated MSCs were characterized (Figure S1 in Supplementary Materials) and expanded under Good Manufacturing Practice (GMP) conditions at CORESTEM Inc. (Seoul, Korea), according to the guidelines of the International Society of Cellular Therapy [27]. The MSCs were cultured in CSBM-A06 medium (CORESTEM Inc.) supplemented with 10% (*v*/*v*) heat-inactivated fetal bovine serum (FBS) (Invitrogen, Carlsbad, CA, USA), 100 U/mL penicillin, 100 μg/mL streptomycin (Invitrogen), and 2.5 mM l-alanyl-l-glutamine (Gibco, Gran Island, NY, USA). The cells were maintained in a humidified atmosphere of 5% CO_2_ in an incubator at 37 °C.

#### 2.1.2. Glial Cell Culture

BV-2 mouse microglial cells were maintained in Dulbecco’s modified Eagle medium (DMEM) supplemented with 5% (*v*/*v*) FBS and gentamicin (50 μg/mL) (Lonza, Basel, Switzerland) in a humidified atmosphere of 5% CO_2_ in an incubator at 37 °C. For primary culture of the glial cells, neonatal astrocyte culture was prepared from mixed glial cultures, as described previously, with minor modifications [28]. Briefly, whole brains from 3-day-old C57BL/6 mice were chopped and were mechanically disrupted using a nylon mesh. The cells obtained were seeded in culture flasks and grown at 37 °C in a 5% CO_2_ atmosphere in DMEM supplemented with 10% heat-inactivated FBS, 100 U/mL penicillin, and 100 μg/mL streptomycin. The culture medium was changed initially after 5 days and then every 3 days. Cells were used after 14–21 days of culture. Primary astrocytes were obtained by shaking mixed glial cultures at 250 rpm overnight. To determine the degree of microglial contamination in the primary astrocyte cultures, traditional reverse transcription polymerase chain reaction amplification of microglia or myeloid markers, such as ionized calcium-binding adapter molecule 1 (Iba-1), Cx3cr1, F4/80, CD11c, and CD11d, was performed [29]. The purity of the astrocyte culture was determined by GFAP immunocytochemistry, as previously described [30]. Primary microglia were obtained by mild trypsinization of mixed glial cultures [31].

#### 2.1.3. Primary Cerebellar Granule Neuron Culture

Cerebellar granule neurons were prepared from mice at embryonic day 16.5, as described previously [32]. Briefly, mouse embryos were decapitated, and their brains were removed rapidly and placed in a culture dish containing cold Hanks’ balanced salt solution (HBSS) supplemented with sodium pyruvate (Gibco) and HEPES (Gibco). The cerebellum was isolated and transferred to a conical tube containing 0.25% trypsin-EDTA for 30 min at 37 °C. Cerebellar tissue was dissociated mechanically by gentle pipetting, and the resulting dissociated cerebellar granule neuronal cells were seeded onto plates coated with poly-d-lysine (Sigma-Aldrich, St. Louis, MO, USA) in neurobasal medium containing 2 mM glutamine (Sigma-Aldrich, MO, USA), penicillin–streptomycin, N2 supplement (Invitrogen, CA, USA), and B27 supplement (Invitrogen, CA, USA).

### 2.2. Preparation of Conditioned Media from MSC Cultures

MSCs at passage 3 were cultured to 70–80% confluence in uncoated plastic culture dishes (Corning, NY, USA). The cells were then washed thoroughly 5 times using 10 mL of HBSS without Ca^2+^ and Mg^2+^, and serum-free CSBM-A06 was replenished. After 74 h, conditioned medium from MSC culture (MSC-CM) was harvested and centrifuged at 500× *g* for 10 min at 4 °C to remove cell debris and was then frozen in aliquots at −80 °C.

### 2.3. Cell Proliferation Assay

Cells were seeded at a density of 1 × 10^3^ cells/well in IncuCyte^®^ ImageLock plates (Essen Bioscience, Ann Arbor, MI, USA). After 24 h, real-time images of cell confluence were acquired every 2–3 h, using the IncuCyte ZOOM Live-Cell Imaging System (Essen Bioscience, MI, USA). Cell proliferation was quantified according to the time-lapse curves generated by the IncuCyte ZOOM software (Essen Bioscience, MI, USA).

### 2.4. Cell Migration Assay

An in vitro wound healing assay was performed using the IncuCyte ZOOM Live-Cell Imaging System (Essen Bioscience, MI, USA). Cells were seeded at a density of 2.5 × 10^4^ cells/well in IncuCyte^®^ ImageLock plates (Essen Bioscience). The cells were then treated with mitomycin C (5 μg/mL) for 2 h before introduction of a wound to inhibit cell proliferation. Wounds were made using the IncuCyte WoundMaker™ (Essen Bioscience), and plates were automatically analyzed for wound closure, using the IncuCyte ZOOM Live-Cell Imaging System (Essen Bioscience). Real-time images were acquired every 2–3 h for 48 h. Cell confluence was quantified using time-lapse curves generated by the IncuCyte ZOOM software (Essen Bioscience).

### 2.5. Nitrite Quantification and Assessment of Cell Viability

Nitric oxide (NO) production was assessed by measuring the amount of nitrite, as previously described [33]. Briefly, after 24-h incubation, 50 μL of cell culture medium was mixed with an equal volume of Griess reagent (0.1% naphthylethylenediamine dihydrochloride and 1% sulfanilamide in 5% phosphoric acid) in a 96-well microtiter plate. Absorbance was measured at 540 nm on a microplate reader. Sodium nitrite was used to create a standard curve for the calculation of the NO concentration. Cell viability was measured using the 3-(4,5-dimethylthiazol-2-yl)-2,5-diphenyltetrazolium bromide (MTT) assay, as previously described [34]. MTT (0.5 mg/mL in phosphate-buffered saline [PBS]) was added to the cells, which were incubated at 37 °C for 2 h in a 5% CO_2_ incubator. The insoluble formazan crystals were then completely dissolved in DMSO, and the absorbance was measured at 570 nm using a microplate reader.

### 2.6. Secretome Analysis

#### 2.6.1. Peptide Generation Using In-Solution Digestion

For in-solution digestion, the precipitated proteins were resuspended in 40 mM NH_4_HCO_3_. After 20-min incubation with 5 mM dithiothreitol at 56 °C, protein precipitates were treated with 10 mM iodoacetamide for 15 min in the dark at room temperature. Then, the protein precipitates were treated with 1:100 trypsin–Lys C mixture (Promega, Madison, WI, USA) for 12 h at 37 °C. Tryptic digested peptides were lyophilized and desalted using a desalting column (#89873, Thermo Fisher Scientific, San Jose, CA, USA), according to the manufacturer’s protocol.

#### 2.6.2. Mass Analysis and Database Search

Tryptic digested peptides were analyzed using a Q-Exactive plus hybrid quadrupole orbit-trap mass spectrometer (Thermo Fisher Scientific) interfaced with an EASY-Spray source. Chromatographic separation of peptides was achieved on an Ultimate 3000 RSLCnano System (Thermo Fisher scientific) equipped with Acclaim PepMap^TM^ 100 (75 mm × 2 cm, 3 µm, nanoViper, Thermo Fisher Scientific) as the loading column and EASY-Spray column PepMap^TM^ RSLC C18 (75 µm × 50 cm, 2 µm, Thermo Fisher Scientific) as the separation column. Peptides were loaded from an RS auto-sampler and separated with a linear gradient of ACN/water, containing 0.1% formic acid, with a flow rate of 300 nL/min. The LC eluent was electrosprayed directly from the analytical column, and a voltage of 2.0 kV was applied via the liquid junction of the nanospray source. Peptide mixtures were separated with a gradient from 5% to 40% ACN in 40 min. The analysis method involved a full mass spectrometry (MS) scan with a range of 350 to 2000 *m*/*z* and data-dependent MS/MS (MS2) on the 10 most intense ions from the full MS scan. The mass spectrometer was programmed to acquire data in the data-dependent mode. Calibration of the mass spectrometer was performed with the proposed calibration solution, according to the manufacturer’s instructions. To perform a database search, tandem mass spectra were processed with Proteome Discoverer software version 2.3 (Thermo Fisher Scientific). The spectral data were searched against the human Uniprot database (release version 2019_07). The analysis workflow used included four nodes, namely, Spectrum Files (data input), Spectrum Selector (spectrum and feature retrieval), Sequest HT (sequence database search), and Percolator (peptide spectral match or PSM validation and FDR analysis). All identified proteins had a FDR of ≤1%, which was calculated at the peptide level. Validation was based on the q-value. Search parameters allowed for tryptic specificity of up to two missed cleavages, with methylthio modification of cysteine as a fixed modification and oxidation of methionine as a dynamic modification. The mass search parameters for +1, +2, and +3 ions included mass error tolerances of 20 ppm for precursor ions and 0.6 Da for fragment ions.

### 2.7. Traditional RT-PCR

Total RNA was extracted from cells using the QIA-zol reagent (QIAGEN, Valencia, CA, USA), according to the manufacturer’s instructions. Reverse transcription and PCR amplification were performed using a DNA Engine Tetrad Peltier Thermal Cycler (MJ Research, Waltham, MA, USA) with specific primer sets (Table 1). Gapdh was used as an internal control. The nucleotide sequences of the primers were based on published cDNA sequences.

### 2.8. Enzyme-Linked Immunosorbent Assay

BV-2 microglial cells or primary microglia cultures were treated with lipopolysaccharide (LPS) and MSC-CM. After 24- or 48-h incubation, the levels of TNF-α in microglia culture media were measured using rat monoclonal anti-mouse TNF-α antibodies (1:180; R&D Systems, Minneapolis, MN, USA) as capture antibodies and goat biotinylated polyclonal anti-mouse TNF-α antibodies (1:180; R&D Systems) as detection antibodies. The biotinylated anti-TNF-α antibodies were detected by sequential incubation with streptavidin-horseradish peroxidase (HRP) conjugate (1:120; R&D Systems) and 3,3′,5,5′-tetramethylbenzidine (TMB) substrates (R&D Systems). After 20-min incubation, the reaction was stopped by adding 2 N H_2_SO_4_, and the absorbance was read at 450 and 540 nm using a microplate reader.

### 2.9. Statistical Analysis

Data are presented as mean ± SEM or SD. Comparisons of two groups were carried out using an unpaired two-tailed *t*-test or ordinary one-way analysis of variance (ANOVA) followed by the Tukey post-hoc test. All statistical analyses were performed using Prism software version 8.0 (GraphPad Software, San Jose, CA, USA). A *p*-value < 0.05 was considered statistically significant.

## 3. Results

### 3.1. Cell Morphology, Proliferation, and Migration

**Figure 1:**
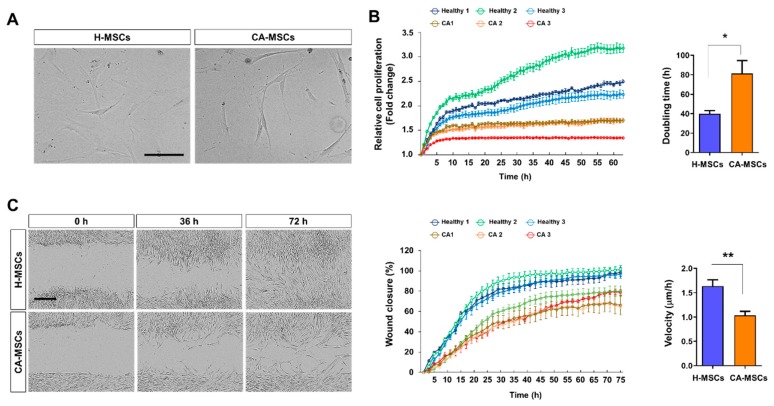
Comparison of the biological characteristics of mesenchymal stem cells (MSCs) derived from cerebellar ataxia patients (CA-MSCs) and healthy individuals (H-MSCs). (**A**) Comparison of the morphology of H-MSCs and CA-MSCs. Scale bar = 200 μm. (**B**) Comparison of the proliferation of H-MSCs and CA-MSCs. The cell proliferation rate was assessed using the IncuCyte Time-lapse Microscopy System and was presented as fold change of cell density. Right panel shows doubling time of MSCs. (**C**) Scratch-wound healing assay. Representative bright-field images showing the migration potential of MSCs at 0, 36, and 72 h after wound scratch. Scale bar = 400 μm. In vitro cell migration and wound closure were assessed every 2 h. The scratch gap was calculated as a percentage of the relative cell density based on the initial wound area (middle panel). Right panel shows the velocity (μm/h) of wound closure. Error bars indicate the standard deviation of three replicate experiments. Data are presented as mean ± SEM. One-way analysis of variance was followed by the Tukey post-hoc test. * *p* < 0.05, ** *p* < 0.01.

We first characterized morphological differences of mesenchymal stem cells (MSCs) derived from cerebellar ataxia patients (CA-MSCs) and healthy individuals (H-MSCs). MSCs were cultured for 24 h and imaged using phase-contrast microscopy (Figure 1A). CA-MSCs had a slender and less flattened morphology with fewer cell protrusions when compared with the findings of H-MSCs. Because of the importance of MSC proliferation and cell migration in therapeutic applications, such as culture expansion and homing [35], we analyzed the proliferative potential and migration capacity of MSCs. The proliferative potential of MSCs was evaluated by detecting the fold change of time-dependent cell density compared with the initial cell density, as well as cell doubling time. As indicated in Figure 1B, the proliferative capacity of CA-MSCs was lower than that of H-MSCs. Subsequently, we quantified the migration of MSCs using a scratch-wound healing assay (Figure 1C). The migration capacity and velocity of CA-MSCs were significantly decreased when compared with those of H-MSCs. These results demonstrate that the potential of proliferation and migration is lower in CA-MSCs than in H-MSCs.

### 3.2. Immunomodulatory Potential of MSCs against Glial Activation

**Figure 2:**
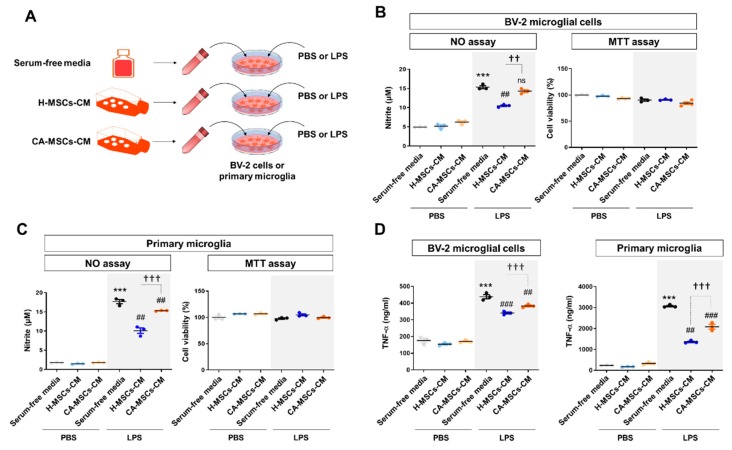
Characteristics of the immunomodulatory potential of MSCs derived from CA-MSCs on microglial activation. (**A**) Schematic drawing of the experimental procedure. The conditioned medium obtained from MSCs culture (MSCs-CM) was added with/without lipopolysaccharide (LPS) (100 ng/mL) to BV-2 microglial cells or primary microglia for 24 h. (**B**,**C**) Nitric oxide assay in BV-2 cells and primary microglia cell culture was performed in triplicate. Nitric oxide production was measured using Griess reagents and was used to indicate microglial activation. (**D**) TNF-α production in BV-2 cells and primary microglia cell culture was measured in triplicate. The TNF-α level was measured using an enzyme-linked immunosorbent assay. Results are presented as mean ± SEM obtained from MSC culture (MSC-CM) from healthy subjects (*n* = 3) or CA patients (*n* = 3). One-way analysis of variance was followed by the Tukey post-hoc test. *** *p* < 0.001 vs. control; ^##^ *p* < 0.01, ^###^ *p* < 0.001 vs. control + LPS; ^††^ *p* < 0.01, ^†††^ *p* < 0.001 vs. healthy MSC-CM + LPS. ns, not significant.

Anti-inflammatory and immunomodulatory activities of MSCs have been reported in vitro and in vivo. We compared the immunomodulatory effect of MSC-CM on glial activation (Figure 2A). BV-2 immortalized murine microglial cells, primary cultured microglial cells, and primary astrocytes were incubated with MSC-CM plus PBS or LPS (100 ng/mL) for 24 h. We observed a significant decrease in NO production in these cells when incubated with H-MSC-CM. However, CA-MSC-CM exhibited no effect or a less immunosuppressive effect than that of H-MSC-CM (Figure 2B,C; Figure S2). In addition, H-MSC-CM reduced LPS-induced TNF-α production in microglial cells, whereas CA-MSC-CM had a less suppressive effect than that of H-MSC-CM (Figure 2D).

### 3.3. Neuroprotective Potential of MSCs

**Figure 3:**
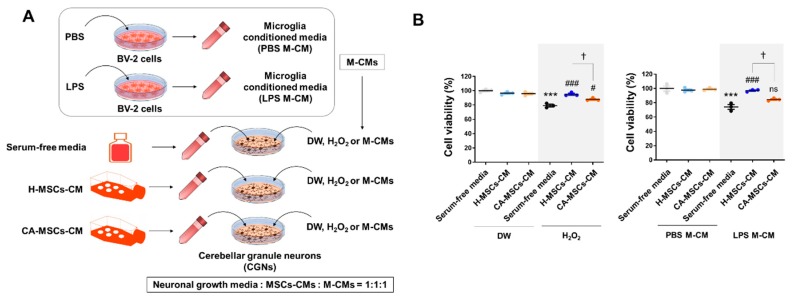
Characteristics of the neuroprotective potential of MSCs derived from CA-MSCs on oxidative stress or microglial toxicity. (**A**) Schematic drawing of the experimental procedure. The conditioned medium obtained from MSC culture was added with/without H_2_O_2_ (200 µM) or microglial conditioned medium (M-CM). M-CM was generated by BV-2 microglial cell culture stimulated with LPS (100 ng/mL) for 24 h. (**B**) Cell viability assay in cerebellar granule neurons (CGNs) was performed in triplicate. Cell viability was measured using the MTT assay. Results are presented as mean ± SEM obtained from MSC-CM from healthy subjects (*n* = 3) or CA patients (*n* = 3). One-way analysis of variance was followed by the Tukey post-hoc test. *** *p* < 0.001 vs. control; ^#^ *p* < 0.05, ^###^ *p* < 0.001 vs. control + H_2_O_2_ or M-CM; ^†^ *p* < 0.05 vs. healthy MSC-CM + H_2_O_2_ or M-CM. ns, not significant. DW, distilled water.

We evaluated whether MSCs have a neuroprotective potential against oxidative stress or microglial toxicity. Addition of exogenous H_2_O_2_ reduced cell viability in cerebellar granule neurons (CGNs), but H-MSC-CM completely rescued the cell viability of CGNs (Figure 3). In addition, we assessed the neuroprotective effect of MSCs on microglia-induced toxicity in CGNs (Figure 3). BV-2 microglial cells were cultured and stimulated by LPS (100 ng/mL) for 24 h. Then, we made a mixture (1:1:1) of neuron growth medium, MSC-CM (serum-free medium or CM of MSCs), and microglial conditioned medium (M-CM) (CM of PBS- or LPS-treated BV-2 cells). The CM of BV-2 cells stimulated by LPS might contain cytokines/chemokines, nucleic acids, excitatory amino acids, reactive oxygen species, and proteases [36]. The mixture of the cell-free normal medium and LPS-stimulated BV-2-CM significantly reduced the cell viability of CGNs, whereas the mixture involving H-MSC-CM rescued the reduced cell viability. The mixture involving CA-MSC-CM also reduced both oxidative stress-induced neurotoxicity and microglia neurotoxicity, but the effect was lower than that of H-MSC-CM.

### 3.4. Comparison of the MSC Secretome

**Figure 4:**
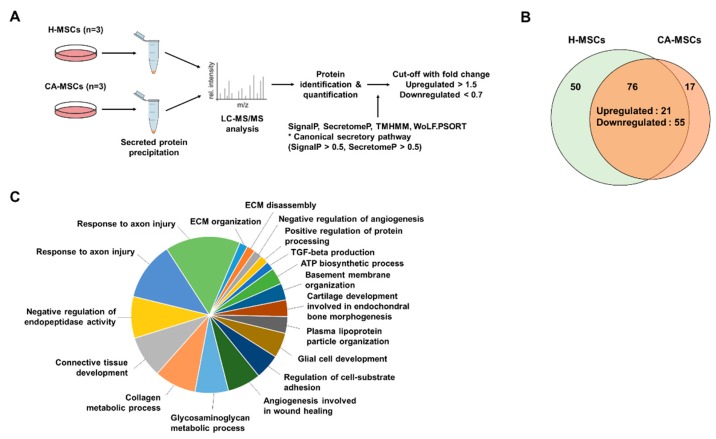
Characteristics of the secretome of MSCs derived from CA-MSCs. (**A**) Schematic drawing of the secretome analysis procedure. (**B**) Venn diagrams showing the number of identified proteins in the secretome of MSCs derived from healthy individuals (H-MSCs) and CA-MSCs. Overall, 50 proteins are exclusively identified in H-MSCs, 17 are identified in CA-MSCs, and 76 are identified in both MSCs. (**C**) Pie chart illustrates the functional analysis using the biological processes of the downregulated secretome in CA-MSCs.

To identify a difference in the secreted proteins between H-MSCs and CA-MSCs, the secretome present in MSC-CM was analyzed according to the protein intensities measured by LC/MS-MS (Figure 4). The MSC-CM was harvested according to the outlined workflow (Figure 4A) at passage 3. A total of 143 different proteins was identified (Supplemental Table S1). In the H-MSC-CM, 50 proteins were uniquely detected and 76 proteins overlapped with CA-MSC-CM. On the other hand, 17 proteins were exclusive to CA-MSC-CM (Figure 4B). To examine the functional distribution in each MSC, we performed gene ontology (GO) enrichment analysis using the DAVID bioinformatics tool. The significance (log *p*-value) of biological process enrichment in each MSC was shown by heat maps (Figure S3). Data revealed that some phenotypes, such as cell migration, cell redox homeostasis, and transforming growth factor-beta receptor signaling pathway, were significantly low in CA-MSCs. Subsequently, we categorized a set of differently detected proteins in MSC-CM among 76 proteins overlapped in the two groups. We focused on the downregulated proteins of CA-MSCs when making comparisons with H-MSCs. We found that 55 proteins had lower protein intensities in CA-MSCs, although these were commonly found in the two groups, and 50 proteins could not be detected in CA-MSCs. As assessed by ClueGO, the functional clusters, such as response to axon injury, negative regulation of endopeptidase activity, glial cell development, and transforming growth factor-beta production, were found to be enriched in the downregulated secretome of CA-MSCs (Figure 4C). Thus, the data indicated a deficiency of the neuroprotective potential of CA-MSCs.

### 3.5. Validation of MSC Secretome Analysis

**Figure 5:**
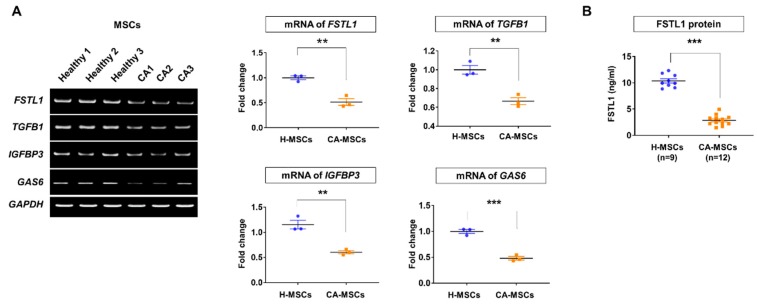
Validation of the secretome analysis results. (**A**) Validation of the identified proteins in secretome analysis according to mRNA expression by reverse transcription polymerase chain reaction. (**B**) Protein validation of FSTL1 in the conditioned media of mesenchymal stem cells (MSCs) derived from H-MSCs (*n* = 9) and MSCs derived from cerebellar ataxia patients (CA-MSCs) (*n* = 12). Data are presented as mean ± SEM. Comparisons of the two groups were carried out using an unpaired two-tailed *t*-test. ** *p* < 0.01, *** *p* < 0.001 vs. H-MSCs.

Among the 55 proteins that were commonly detected in the two groups but were lower in CA-MSCs, we validated the mRNA expressions of randomly selected proteins, such follistatin-like 1 (FSTL1), transforming growth factor-beta 1 (TGFB1), insulin-like growth factor-binding protein 3 (IGFBP3), and growth arrest-specific 6 (GAS6) (Figure 5A). Data revealed that the levels of *FSTL1*, *TGFB1*, *IGBP3*, and *GAS6* mRNA were significantly lower in CA-MSCs than in H-MSCs. We focused on the low level of FSTL1 in CA-MSCs, which is known to be an immune modulator. The validation of the FSTL1 protein in CA-MSC-CM using an enzyme-linked immunosorbent assay showed a lower level than that in H-MSCs (Figure 5B).

### 3.6. FSTL1 Suppresses Inflammatory Activation of Microglia

**Figure 6:**
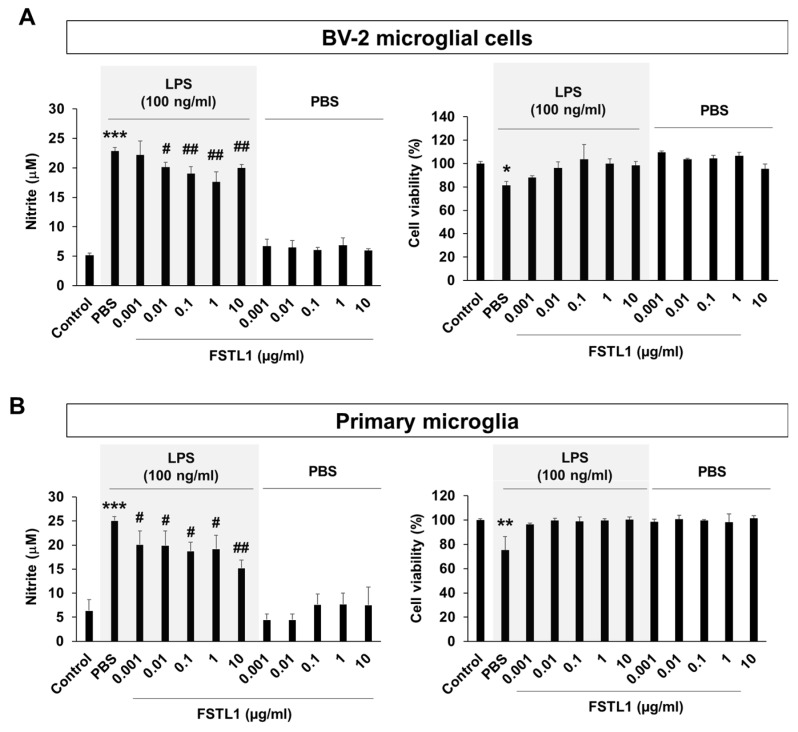
FSTL1 has a suppressive potential for microglial activation. (**A**) FSTL1 inhibited LPS-induced nitric oxide production in BV-2 microglial cells. BV-2 cells were stimulated with LPS (100 ng/mL) for 24 h after 2-h pretreatment with different concentrations of FSTL1 (*n* = 3 each). (**B**) Suppressive effect of FSTL1 on LPS-induced nitric oxide production in primary microglia. Data are presented as mean ± SEM. One-way analysis of variance was followed by the Tukey post-hoc test. * *p* < 0.05, ** *p* < 0.01, *** *p* < 0.001 vs. control; ^#^ *p* < 0.05, ^##^ *p* < 0.01 vs. vehicle control (phosphate-buffered saline, PBS).

To determine whether FSTL1 modulates microglia-mediated neuroinflammation, we administered recombinant FSTL1 protein (dose range from 0.0001 to 10 µg/mL) to BV-2 cells or primary microglial cells before LPS (100 ng/mL) stimulation. The NO assay revealed that addition of FSTL1 protein mitigated LPS-induced microglial activation (Figure 6) in a dose-dependent manner. Our results suggest that the FSTL1 protein in MSC-CM might have an anti-inflammatory role for activated microglia.

## 4. Discussion

We demonstrated the differences in the functional phenotypes of MSCs between H-MSCs and CA-MSCs. CA-MSCs showed low capacity in terms of proliferation, migration, and immune modulation. Comparison of the secretome between H-MSCs and CA-MSCs indicated a lower therapeutic potential of CA-MSCs. The functional clustering of the secretome using bioinformatics analysis tools revealed lower protective functions associated with response to injury, inhibition of protease activity, and angiogenesis in CA-MSCs. In accordance with previous studies [37,38], our findings suggest a limitation of autologous MSC therapy for CA. Recently, the bone marrow microenvironment and cellular functions were reported to be affected by disease states [39,40,41]. Thus, MSCs from patients with chronic diseases may have reprogrammed gene expression profiles. For example, fibroblast growth factor-10 was markedly suppressed in the subjects with progressive idiopathic pulmonary fibrosis, and both TGF-β1 and SHH signaling were identified as critical mediators of this effect in MSCs [39]. MSCs from type 1 diabetes patients presented sympathetic nervous system signaling hyperactivity with an increase of β3-adrenergic receptor gene expression and upregulation of related pathway [40]. Moreover, metabolic syndrome seems to limit therapeutic potential of MSCs. MSCs from type 2 diabetes patients showed upregulated autophagy and mitochondria deterioration that indirectly affect MSC fate [42]. Although underestimated, epigenetic and genetic instability might also affect longevity and regenerative capacity of MSCs [43]. In this study, the secretome analyses revealed the reduced cell proliferation, migration, and anti-inflammatory capacity (TGF-β signaling pathway) in CA-MSCs (Supplemental Figure S2). These defects and molecular alterations may potentially influence the capacity of the CA-MSCs for therapeutic usage. Moreover, MSCs can be primed by preexposure to noxious stimuli [44], implying that prolonged stimulation under pathological conditions might be associated with a loss of the putative disease-ameliorating capacity of MSCs. In addition, the age of donors has been considered as a factor that might limit the effectiveness of MSC-based therapy [45,46], particularly autologous MSC therapy. Senescence affects various functions of MSCs [47,48], reduces the capacity for migration and homing [49], and impairs the immunosuppressive potential [50].

The CA-MSCs in culture presented a senescent phenotype and a reduced defensive potential against oxidative stress and microglial toxicity. In addition, our secretome analysis revealed that approximately 75% of secreted proteins were downregulated in CA-MSCs when compared with the findings in H-MSCs. The downregulated proteins were mainly associated with homeostasis and protection (for example, TGFB1, IGFBP3, GAS6, and FSTL1). The loss of TGFB1 has been shown to promote neuronal death and microgliosis [51]. IGFBP3 has been shown to enhance pericyte ensheathment and microglial apoptosis after ischemic retinal injury [52]. Lack of GAS6 has been found to prolong neuroinflammation, leading to extensive axonal damage, following cuprizone exposure [53]. In the present study, we focused on FSTL1, which is known to be a neuroprotective protein. FSTL1 is a secreted extracellular glycoprotein and was initially identified as a proinflammatory cytokine [54] and named TGF-β-stimulated clone 36 (TSC-36) [55]. FSTL1 belongs to the secreted protein acidic and rich in cysteine (SPARC) family, which contains both extracellular calcium-binding and follistatin-like domains [56], and regulates cell survival, proliferation, differentiation, inflammation, and other essential processes [57]. At present, the role of FSTL1 in inflammation is controversial. FSTL1 has been shown to upregulate proinflammatory cytokines in the liver [54]. However, administration of FSTL1 reduced ischemic damage and inflammatory response with AMP-activated protein kinase- and bone morphogenetic protein-4-dependent mechanisms in a myocardial infarction model [58]. Circulating FSTL1 inhibited the synthesis of pro-inflammatory cytokines, including IL-1β in a cisplatin-induced nephrotoxicity model [59] and TNF-α, IL-6, and monocyte chemotactic protein-1 in a subtotal nephrectomy model [60]. Intravenous administration of an adenovirus expressing FSTL1 reduced the expression of proinflammatory cytokines (IL-17A, IL-6, and IFN-γ) and thereby prolonged the survival of transplanted patients [61]. In the brain, FSTL1 was shown to downregulate the expression of proinflammatory cytokines via suppression of the MAPK/p-ERK1/2 pathway in astrocytes [62]. In contrast, astrocytic FSTL1 has been implicated in polyI:C-induced neurodevelopmental impairment as a proinflammatory molecule [63]. Mattiotti et al. demonstrated that the glycosylation state of FSTL1 is a determinant of biological activity and might be involved in the differences between species and tissues [64]. FSTL1 has three potential sites for *N*-glycosylation and two for *O*-glycosylation. In vitro studies have demonstrated that only the three aspartate residues Asp142, Asp173, and Asp178 are *N*-glycosylated. Glycosylation at these sites was found to be cell type-specific [56]. In the present study, we found that recombinant FSTL1 protein suppressed LPS-induced microglial activation. Thus, it is likely that MSC-derived FSTL1 might have inhibitory effects on microglia-mediated neuroinflammation.

The neuropathology of CA is diverse. CA is characterized by degeneration of the cell bodies and idiodendritic trees of Purkinje neurons [65], as well as reactive gliosis in the cerebellum [66,67]. CA shows widely varying age of onset. Sporadic ataxia usually begins in middle age [68]. The investigation based on a large cohort of CA patients demonstrated that CAs are the result of diverse disease processes that can be genetic, gluten ataxia, idiopathic, alcohol-related, and others [23]. Nevertheless, immune-mediated ataxias are frequent. CA has strong immune and inflammatory components [69], and neuroinflammation is considered as an important therapeutic target. Taken together, our findings indicate that CA-MSCs have lower secretory potentials for anti-neuroinflammatory proteins, such as FSTL1, when compared with the findings of H-MSCs. To our knowledge, this is the first study to characterize MSCs isolated from CA patients. Our study findings might have important implications for the establishment of an effective MSC therapy for CA.

## Figures and Tables

**Table 1:** DNA sequences of the primers used for RT-PCR.

Gene	Primer Sequences	GenBank Accession No.
*FSTL1*	F: 5′-CGATGGACACTGCAAAGAGA-3′ R: 5′-TCAGGAGGGTTGAAAGATGG-3′	NM_007085.5
*TGFB1*	F: 5′-CACGTGGAGCTGTACCAGAA-3′ R: 5′-GAACCCGTTGATGTCCACTT-3′	NM_000660.6
*IGFBP3*	F: 5′-ACAGCCAGCGCTACAAAGTT-3′ R: 5′-TAGCAGTGCACGTCCTCCTT-3′	DQ301819.1
*GAS6*	F: 5′-ATCAAGGTCAACAGGGATGC-3′ R: 5′-CTTCTCCGTTCAGCCAGTTC-3′	NM_000820.4
*GAPDH*	F: 5′-GAAATCCCATCACCATCTTCC-3′ R: 5′-GAGGCTGTTGTCATACTTCTC-3′	NM_001289745.2

## Supplementary Materials

The following are available online at https://www.mdpi.com/2073-4409/9/1/212/s1: Figure S1: Characterization of MSCs derived from bone marrow. (**A**) Multilineage differentiation capacity of MSCs. MSCs were cultured into 24-well plates and stimulated to differentiate along adipogenic, osteogenic, and chondrogenic lineages using an MSC functional identification kit (R&D Systems, Minneapolis, MN, USA). After differentiation, lipid droplets in adipocytes were visualized with oil red O staining (Sigma, Saint Louis, MO, USA). Osteogenic differentiation was assessed based on the staining of calcium deposits by alizarin red (Sigma, Saint Louis, MO, USA), and chondrogenic differentiation was determined by alcian blue staining (Sigma, Saint Louis, MO, USA). (**B**) Immunophenotypic analysis for expression profiles of cell surface markers of MSCs in the early phases. To confirm the MSC characteristics, cells within five passages were stained with the cell surface markers CD29, CD73, CD90, CD105, CD34, CD45 (BD Pharmingen, Heidelberg, Germany), and CD44 (BD Biosciences, San Diego, CA). The expression of cell surface markers was measured using a flow cytometer (BD FACS Canto™ II), and MSCs were identified as CD29/CD44/CD73/CD105-positive and CD34/CD45-negative cells; Figure S2: Characteristics of the immune modulatory potential of mesenchymal stem cells (MSCs) derived from cerebellar ataxia patients (CA-MSCs) with regard to astrocytes activation. (**A**) Schematic drawing of the experimental procedure. The conditioned medium obtained from MSCs culture was added with/without lipopolysaccharide (LPS) (100 ng/mL) to primary astrocytes for 24 h. (**B**) Nitric oxide assay in primary astrocyte culture. Nitric oxide production was measured using Griess solution and was used as an indicator of glial activation. Data are presented as mean ± SEM. One-way analysis of variance was followed by the Tukey posthoc test. *** *p* < 0.001 vs. control; ^##^ *p* < 0.01, ^###^ *p* < 0.001 vs. control + LPS; ^††^ *p* < 0.01 vs. healthy-MSC-CM + LPS; Figure S3: The *p*-value heat map of significant biological processes within either the secretome of MSCs derived from healthy individuals (H-MSCs) or MSCs derived from CA-MSCs. The heat map is colored by the significance (log *p*-value) of enrichment of the biological process, where a darker red indicates a higher degree of enrichment and darker green indicates a higher degree of deficiency of functional activities. Ontological analysis was performed using the Database for Annotation, Visualization, and Integrated Discovery (DAVID, version 6.8); Table S1: List of downregulated proteins in the secretome analysis; Table S2: Functional groups analyzed by ClueGO.
